# Wobble-Board Dynamics Identify Individual Signatures of Balance Control for Clinical Assessment

**DOI:** 10.1007/s10439-025-03955-0

**Published:** 2026-01-02

**Authors:** Theodoros Deligiannis, Madhur Mangalam

**Affiliations:** Department of Biomechanics, University of Nebraska at Omaha, 6160 University Drive S, Omaha, NE 68182 USA

**Keywords:** Balance, Center-of-mass, Center-of-pressure, Coordination, Fractal, Postural control

## Abstract

**Purpose:**

Understanding balance control mechanisms is essential for designing effective assessment and rehabilitation strategies. Wobble-board tasks that introduce continuous postural instability offer a valuable method to investigate and enhance proactive and emergent balance control mechanisms. This study aimed to investigate proactive and emergent balance control strategies using a mediolaterally unstable wobble board, focusing on several spatial and temporal characteristics of balance control in healthy young adults.

**Methods:**

Twenty-nine healthy young adults performed wobble-board tasks under two conditions: with and without a concurrent cognitive load (Trail Making Task). The study examined two primary outcome measures: (i) spatial—peak excursion magnitude of the board and (ii) temporal—cycle duration of the board’s movement. The Hurst exponent was used to characterize the stochastic properties of each series. In addition, Principal Component Analysis (PCA) and Canonical Correlation Analysis (CCA) were employed to assess the relationship between wobble-board dynamics and traditional balance metrics (center-of-pressure, CoP, center-of-mass, CoM, and CoP–CoM).

**Results:**

Temporal control, indexed by cycle duration, exhibited a Hurst exponent near $H\!=\!0.48$, consistent with uncorrelated, emergent adjustments (Brownian-like), whereas spatial control, indexed by excursion magnitude, showed $H\!=\!0.73$, indicating persistent, proactive regulation likely mediated by proprioceptive/reflexive feedback. These signatures were preserved under cognitive load, suggesting robust spatial control alongside load-resilient, self-organizing temporal dynamics. PCA and CCA further showed that the WB metrics capture information complementary to CoP and CoP–CoM, with high cross-set correlations underscoring their multidimensional value. Finally, cyclical WB metrics (e.g., RMS peak angular excursion, cycle frequency) demonstrated moderate-to-high test–retest reliability (ICCs $=\!0.77\text {--}0.86$), whereas fractal-like measures (Hurst exponents) showed lower consistency (ICCs $=\!0.19\text {--}0.45$), indicating greater sensitivity to trial-to-trial fluctuations.

**Conclusion:**

Wobble-board assessment isolates a dual-mode balance strategy in which spatial regulation is actively controlled while temporal structure self-organizes. The separation yields actionable targets for metric-guided, progressive training that emphasizes excursion control while monitoring temporal adaptability. Because these metrics complement conventional CoP/CoM measures, they offer a practical bridge from laboratory to clinic-friendly evaluation and feedback. Validation in aging and clinical cohorts and outcomes-focused longitudinal trials will determine their translational value for fall prevention and rehabilitation.

## Introduction

Understanding the mechanisms of balance control is essential for advancing the assessment and rehabilitation of functional postural stability and fall prevention, particularly as age-related declines in balance significantly increase the risk of falls and associated injuries. Effective balance control relies on the seamless integration of sensory inputs and motor outputs, enabling individuals to maintain stability in response to predictable and unpredictable perturbations [1–12]. Traditional metrics, such as those derived from center-of-pressure (CoP) and center-of-mass (CoM), have been widely used to evaluate balance [13, 14]; however, these measures are primarily suited for static or quasi-static conditions and often require laboratory settings. Consequently, they may not fully capture the dynamic and adaptive capabilities required for real-world tasks or provide practical, clinic- and community-friendly options for ongoing balance improvement. Given the growing need for balance assessment and training in aging populations, there is a critical demand for affordable, accessible approaches that extend beyond the confines of specialized laboratories. Developing and deploying methods that facilitate balance evaluation and enhancement in clinic and community settings is essential to meet the increasing global burden of falls. These approaches must address the complexities of real-world stability demands to promote broader access to effective interventions and mitigate disparities in balance-related healthcare.

Wobble boards, also known as balance boards, are widely used rehabilitation and training devices consisting of a rigid platform mounted on an unstable base that permits uni- or multi-directional tilting movements through a mechanically defined range. Training with these versatile devices has proven effective in improving balance, proprioception, and neuromuscular control across diverse populations. They have been utilized by both athletes and non-athletes aiming to improve postural performance [15–17], as well as those recovering from lower extremity injuries, including stage-2 ankle sprains [18] and ankle instability [19–23]. These devices have also benefited older adults [24, 25] and individuals with neurological conditions, such as stroke survivors seeking to regain balance [26, 27]. Research has consistently demonstrated the clinical benefits of wobble-board training in improving postural stability by reducing postural sway [28–31] across these populations. However, despite their widespread clinical adoption, there remains a fundamental gap in our understanding of balance control mechanisms and strategies employed during wobble-board exercises. The control strategies used to maintain balance on these unstable surfaces have not been fully characterized, leading clinicians to rely primarily on empirical observations rather than evidence-based principles when prescribing wobble-board interventions. A deeper understanding of how individuals control board movements and which aspects of this control most directly influence functional improvement would transform our ability to optimize wobble-board design and implementation. This knowledge is crucial for developing wobble boards with targeted mechanical properties that specifically challenge impaired balance control mechanisms, enabling clinicians to create more effective, personalized rehabilitation protocols based on individual postural control deficits.

Studies suggest that wobble boards can also be utilized as affordable postural assessment devices, offering a practical alternative to expensive force plates and motion tracking systems. Research validating a new measurement approach with an instrumented wobble-board demonstrated strong correlations, $r\!=\!0.66\text {--}0.95$, between the board’s kinematic measurements and traditional CoP data from force platforms [32]. This device proved valid and reliable for assessing dynamic balance in young adults, with excellent test consistency both within sessions ICC$\approx \!0.89\text {--}0.95$ and between sessions ICC$\approx \!0.66\text {--}0.95$. Similarly, evaluations of a computerized wobble board showed fair to excellent reliability between sessions and acceptable error levels, though it exhibited poor correlation with “Y Balance Test” scores [33]. This indicates that while the wobble board may assess different aspects of balance than the Y Balance Test, it remains a reliable and valid standalone device for dynamic balance evaluation. However, information is scarce, and it remains unclear to what extent wobble boards provide the same information, less, or potentially unique insights compared to traditional CoP and CoM-based assessments. This gap underscores the need for rigorous investigations to determine their full potential as reliable, cost-effective devices for postural assessment and training in both clinical and community settings.

The utility of wobble boards as both balance assessment and training devices hinges on a comprehensive understanding of the balance control mechanisms employed when standing on these devices and the intricate dynamics of the body–board system. These dynamics are inherently complex, involving diverse biomechanical and neuromuscular control strategies extending beyond traditional approaches on firm surfaces. Research has demonstrated that balancing on a wobble board requires movement strategies beyond ankle control—the primary mechanism for balance on stable surfaces—indicating a fundamentally different biomechanical approach [34]. Weak and insignificant correlations between CoP metrics on firm surfaces and the same metrics on wobble boards further underscore this biomechanical divergence. Another study, which modeled human balance on a wobble board as an inverted pendulum system, found that small changes in factors such as board stiffness, neuromuscular response strength, and sensory feedback delays can cause abrupt transitions between stable and unstable balance states [35]. Additionally, the analysis revealed the neuromuscular system’s adaptability in achieving stability on wobble boards, characterized by unique patterns of oscillatory and divergent responses in CoP metrics. These findings collectively suggest that wobble boards assess dimensions of balance performance beyond what traditional CoP and CoM metrics can capture, with the board’s inherent properties playing a critical role in shaping these distinctions, highlighting the need for thorough investigations into their dynamics and applications.

Temporal and spatial adaptations contribute distinct yet complementary roles in the learning and execution of movements. Temporal adaptations, such as fine-tuning step durations, are critical for tasks requiring precise timing and rhythm. For example, during split-belt treadmill walking, adjustments in step timing rapidly restore gait symmetry, highlighting robust and stable neural pathways dedicated to temporal coordination [36]. In contrast, spatial learning emphasizes exploration and feedback-driven refinement, as seen in activities like rowing, where iterative adjustments to movement patterns improve performance [37]. Evidence suggests that these processes may operate independently: spatial adaptations often rely on flexible, context-dependent frameworks, while temporal adaptations depend on stable, time-driven rhythms [38]. This distinction underscores the importance of balancing spatial flexibility with precise temporal control in optimizing movement rehabilitation and performance training. Building on this understanding, the present study seeks to extend these insights to balance training by independently analyzing temporal and spatial parameters during wobble-board exercises.

To address these concerns, we examined the wobble-board control strategies in $N\!=\!29$ healthy young adults balancing on a mediolaterally aligned wobble board. Our central question was whether individuals deploy distinct strategies for different aspects of balance, differentiating among proactive (anticipatory), reactive (feedback-corrective), and emergent (not directly regulated; arising from task mechanics) mechanisms. We hypothesized that proactively regulated variables would exhibit long-range temporal correlations (Hurst exponent $H\!>\!0.5$) consistent with feedback-based control, whereas variables left emergent would show random fluctuations ($H\!\approx \!0.5$). Accordingly, we predicted higher $H$ in the primary control variable (e.g., mediolateral sway) than secondary variables (e.g., anteroposterior sway), indicating purposeful control in the board’s primary direction of instability. We evaluated these predictions using second-order detrended moving-average analysis (DMA) [39–41] to quantify the spatiotemporal structure of wobble-board motion and to separate controlled from emergent components of balance.

We also examined the robustness of these balance control strategies under concurrent task conditions by introducing a concurrent body-sized trail making task (TMT), where participants had to trace a sequence through projected numbers using a laser pointer. The central question was whether this concurrent task, which demands cognitive processing and upper-body movement, would interfere with the spatiotemporal structure of the wobble-board control. We hypothesized that the concurrent task condition would reveal potential interference between postural and task-oriented control strategies. Specifically, we predicted that if the tasks competed for similar control resources, we would observe a shift in the wobble-board dynamics characterized by reduced long-range correlations (Hurst exponents approaching $0.5$), indicating a breakdown in the organized control structure. Ultimately, the outcomes of this manipulation carry significant implications for refining wobble-board intervention protocols and improving fall prevention strategies, particularly for real-world scenarios that demand simultaneous balance control and precise upper-body coordination.

Understanding how individuals control balance during dynamic tasks requires examining stable, individual-specific strategies and trial-to-trial variability. The cyclical dynamics of balance tasks, such as wobble-board exercises, are hypothesized to reflect consistent, individual-specific “signatures” of postural control—akin to individual-specific signatures in walking [42, 43]. In contrast, more fractal-like measures, such as the Hurst exponent, which captures long-range temporal correlations, may better capture variability across trials. We posed two key questions to test this: (i) Do cyclical metrics of wobble-board dynamics, such as root mean square (RMS) of peak angular excursion and cycle frequency, exhibit high consistency across trials and conditions? (ii) Are fractal-like measures, such as Hurst exponents, more sensitive to fluctuations in responses to wobble-board instability and thus less consistent across trials? We hypothesized that cyclical metrics of wobble-board dynamics would demonstrate moderate-to-high consistency, reflecting stable control strategies, while fractal-like metrics would reveal greater trial-to-trial variability. To address these questions, we computed intraclass correlation coefficients (ICCs) to quantify the stability of repeated measurements relative to overall variability. Based on our predictions, stable cyclical metrics would support the notion of robust, person-specific control, while the lower ICCs of fractal-like measures would highlight their sensitivity to dynamic fluctuations in motor behavior.

Finally, we addressed whether the WB metrics, derived from a dynamic and functional balance task, overlap with traditional CoP and CoP–CoM metrics or provide unique insights into balance control. We hypothesized that the WB metrics would share substantial overlap with traditional metrics, reflecting shared underlying balance mechanisms. However, we further predicted that wobble-board metrics would capture additional, functionally relevant information about balance performance, particularly in dynamic and concurrent task conditions. This additional information would highlight the ability of the WB metrics to assess nuanced aspects of postural control and adaptability that may not be fully captured by CoP and CoP–CoM metrics. Moreover, we anticipated that wobble-board metrics would exhibit sensitivity to individual differences and variability in balance strategies. If this hypothesis were correct, we would expect to find systematic relationships between wobble-board dynamics and traditional laboratory-based metrics. This finding would have significant practical implications, potentially enabling instrumented wobble boards as cost-effective and portable alternatives for balance assessment and training in clinical and community settings. Their ability to capture dynamic and functional aspects of balance control would make them especially suited for diverse applications, ranging from rehabilitation to athletic training.

## Methods

### Participants

This study initially recruited $48$ healthy young adults who voluntarily participated after providing written and verbal informed consent, although data from only $29$ participants (mean age: $25.0\!\pm \!4.1$ years; $16$ women) were included in the present analysis, as the remaining $19$ participants did not have retroreflective markers attached to the wobble board for motion tracking.

### Tasks, Procedure, and Instructions to Participants

**Fig. 1:**
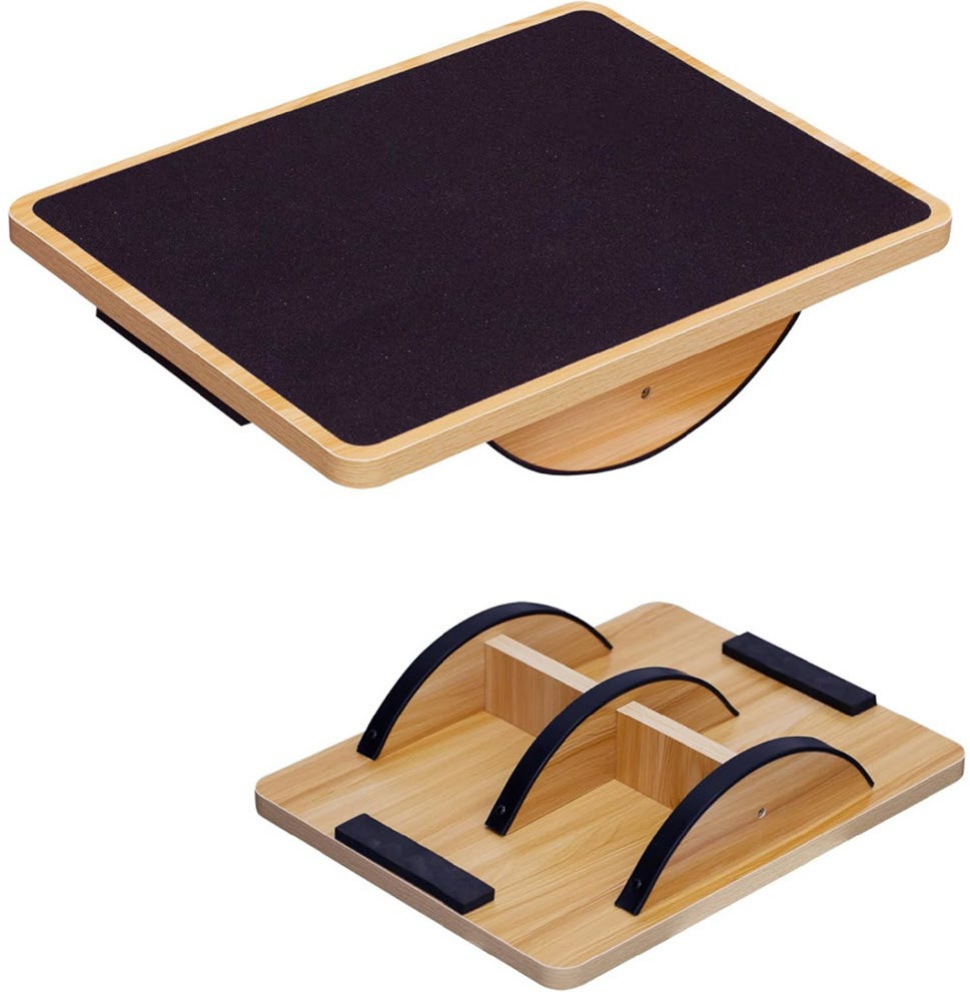
The wobble board used to impose postural instability along the ML axis

The experiment was conducted in the Biomechanics Research Building at the University of Nebraska at Omaha. The wobble board was constructed of wood, with dimensions of $44.5\!\times \!35.0\!\times \!9.5$ cm and fitted with an anti-slip surface, was utilized during the study (Fig. 1). The TMT Part A task was displayed on a $1.80\!\times \!1.80$ m screen, where participants were instructed to follow a path through a randomized sequence of numerical targets. Each participant completed two experimental conditions, with two trials per condition, resulting in a total of four trials. To mitigate potential order effects, the trial order was randomized for every participant. (Note: Two additional conditions without the wobble board with two trials each were conducted as part of the larger study [44–47] but are not included in the present analyses.)

**Fig. 2:**
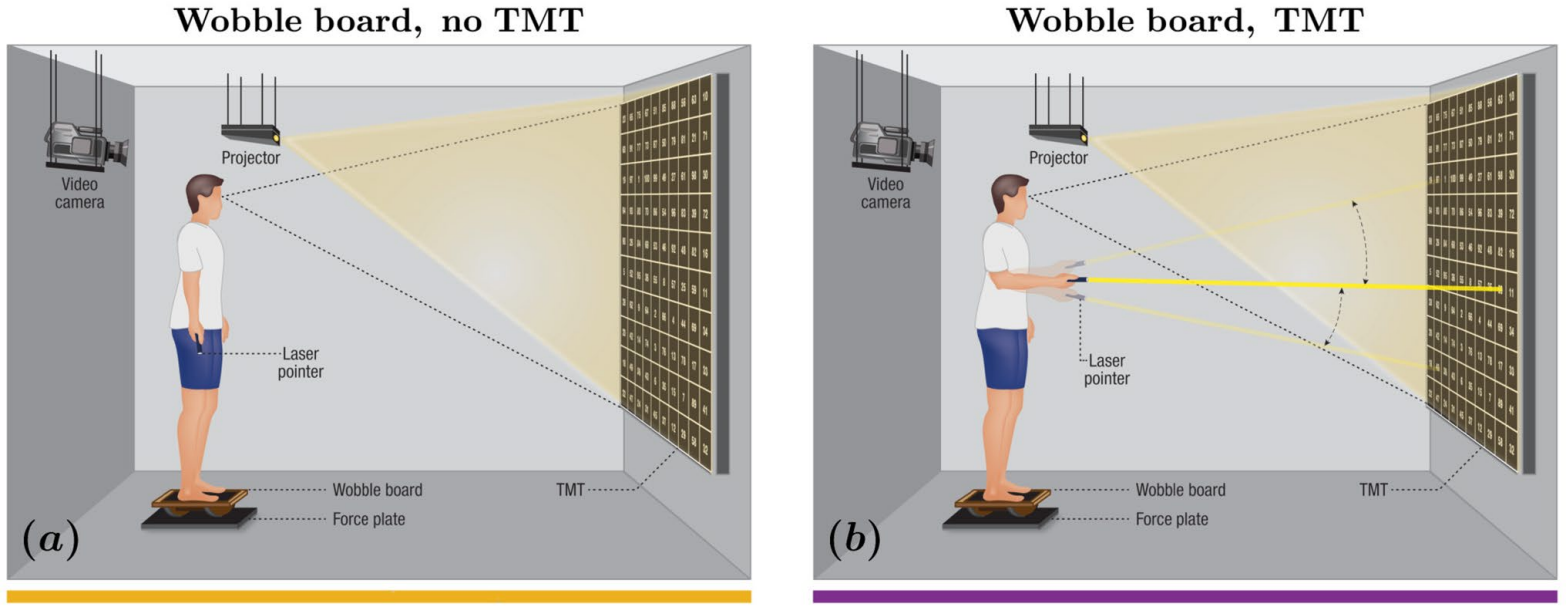
Experimental setup. We combined a body-sized trail making test (TMT) with stabilography and full-body motion tracking. Participants performed two tasks while standing on a wobble board atop a force plate designed to introduce mediolateral instability. They maintained balance on the wobble board in the first condition (**a**). In the second condition (**b**), they used a handheld laser pointer to trace a path connecting randomly placed numbers projected onto a screen. In a third condition (not shown here), participants performed simple standing directly on the force plate without the wobble board to serve as a baseline comparison

In the “wobble board, no TMT (WBNT)” condition (Fig. 2a), participants stood on the wobble board oriented mediolaterally and positioned on a force plate for five minutes. In the “wobble board, TMT (WBWT)” condition (Fig. 2b), participants stood on the wobble board while completing the TMT using a laser pointer. They were instructed to prevent the board’s edges from contacting the force plate; no further guidance, such as minimizing sway, was provided to preserve natural postural control, as even simple instructions can shift attention and alter balance strategies [48]. A unique randomized sequence of numbers was generated for each TMT trial to reduce learning effects across trials, and the TMT was displayed in the WBNT condition to ensure consistency. TMT performance was assessed as the total number of consecutive numbers correctly traced during the trial; higher scores indicate better performance.

### Stabilography

Participants stood either directly on a floor-integrated force plate or on a wobble board positioned atop a $40\!\times \!60$ cm force plate (400600HPS^TM^, AMTI, Watertown, MA). The force plate, equipped with strain gauge transducers, recorded ground reaction forces and moments generated by the participants’ feet at a sampling frequency of $1000$ Hz. The collected ground reaction forces and moments were subsequently used to compute the postural CoP along the ML and AP axes.

### Motion Tracking

For data collection, participants wore form-fitting or exercise clothing and remained barefoot. Twenty-nine retroreflective markers were affixed to specific anatomical landmarks on each participant. This marker set is widely used in biomechanics research due to its standardized placement, which has been extensively validated for both research and clinical applications. The design also optimizes coverage of key anatomical landmarks with a reduced number of markers, minimizing participant discomfort and the likelihood of interference during dynamic movements. Additionally, four markers were placed at the corners of the wobble board to track its movement during trials. During calibration of the whole-body biomechanical model, participants stood on the force plate with their feet apart and assumed a “T” pose, arms abducted approximately $90 ^\circ$, maintaining this position for 5–7 s. Quality control checks indicated inconsistent T-pose alignment in these participants, which could introduce systematic bias into biomechanical model scaling and joint axis orientation estimates. To enforce a standardized neutral posture across all participants, we substituted these T-pose trials with a 7-second segment taken from the start of each participant’﻿s first “no wobble board, no TMT” (NBNT) experimental trial. Marker trajectories were captured in three dimensions at $100$ Hz using a $12$-camera motion capture system (Kestrel 4200^TM^, Motion Analysis, Rohnert Park, CA).

### Data Processing

#### Postural Center-of-Pressure (CoP)

The ground reaction forces and moments recorded by the force plate were used to compute postural CoP along the AP and ML axes using the formula:

$$\mathrm{CoP}_{\mathrm{ML}}(t)=\frac{M_y(t)}{F_z(t)}+\mathrm{origin}^{\mathrm{plate}}_x,\qquad \mathrm{CoP}_{\mathrm{AP}}(t) = -\frac{M_x(t)}{F_z(t)}+\mathrm{origin}^{\mathrm{plate}}_y$$

where $\mathrm{CoP}_{\mathrm{ML}}(t)$ and $\mathrm{CoP}_{\mathrm{AP}}(t)$ are the coordinates of the CoP along the ML and AP axes at timepoint $t$ and $M_{\mathrm{x}}(t)$, $M_{\mathrm{y}}(t)$, and $F_{\mathrm{z}}(t)$ are directional components of the moments and forces acting on the body from the force plate.

#### Postural Center-of-Mass (CoM)

Body-segment geometry and mass properties were defined with the Dempster model for segment mass distribution and orientation [49] and the Hanavan model for segment inertias [50]. Hip joint centers were estimated using Bell’s geometric method from the relative positions of the anterior superior iliac spine (ASIS) markers [51]. Pelvic kinematics were derived from ASIS and sacral markers; shoulder joint centers were defined from bilateral acromial markers. Elbow, wrist, knee, and ankle joint centers were identified using paired medial﻿–lateral markers and the axial offset method following standard Visual3D® (C-Motion, Germantown, MD) procedures; only lateral markers were available for the elbow and wrist due to constraints of the Helen Hayes model. Thorax modeling incorporated iliac landmarks from the Terry database as described by Kepple et al. [52]. All marker processing, model definitions, and computations were performed in Visual3D^®^, yielding whole-body CoM trajectories for each standing trial.

#### Filtering

CoP trajectories along the AP and ML axes were processed independently using a Butterworth filter with a cutoff frequency of $60$ Hz. This filter was selected due to its maximally flat frequency response, which minimizes distortions and ripples, thereby preserving the integrity of the signal [53]. To ensure uniformity across data streams, CoP trajectories were downsampled to $100$ Hz to match the sampling rate of the CoM data. For CoM trajectories, a Butterworth filter with a $6$ Hz cutoff frequency was applied, allowing for the retention of slower, larger-scale movements while effectively attenuating higher-frequency noise. Filtering was performed using a zero-phase low-pass Butterworth filter implemented with MATLAB’s filtfilt() function (MATLAB 2024a, MathWorks, Natick, MA). The filter was designed with the butter() function, and its order was optimized using the buttord() function. Design parameters included a passband edge frequency $W_n$, a stopband edge frequency $W_n\!+\!0.1$, a passband ripple of $3$ dB, and a stopband attenuation of $40$ dB. CoP was computed from the ground reaction forces and moments, while CoM was derived from marker positions prior to filtering to ensure data integrity and avoid distortions introduced by preprocessing [54]. This methodology, which avoids filtering ground reaction forces, moments, or marker trajectories prior to CoP and CoM calculation, is particularly crucial for dynamic activities such as wobble-board tasks.

#### CoP and CoP–CoM Metrics

The metrics summarized in Table 1 were computed to quantify various aspects of CoP, including spatial dispersion, temporal variability, and dynamic characteristics, enabling a comprehensive analysis of postural stability and control. The metrics in Table 2 were calculated to evaluate the dynamic and spatial relationships between the CoP and CoM, offering insights into postural control strategies during balance tasks. Many of these metrics are widely used in biomechanics research [13, 14] due to their ability to provide standardized and interpretable measures of postural performance.

**Table 1:** Center-of-pressure (CoP) metrics used in this study

Metric	Formula	Description
Path length (PL)	$\displaystyle \mathrm{PL} = \sum _{i=1}^{n-1} \sqrt{(x_{i+1} - x_i)^2 + (y_{i+1} - y_i)^2}$	Total distance traversed by the CoP trajectory over the trial
Sway area ($A_{\mathrm{sway}}$)	$\displaystyle A_{\mathrm{sway}} = \frac{1}{2} \Big | \sum _{i=1}^n (x_i y_{i+1} - y_i x_{i+1}) \Big |, \quad (x_{n+1}, y_{n+1}) = (x_1, y_1)$	Area enclosed by the (ordered) CoP path using the shoelace formula
Confidence ellipse area (CEA)	$\displaystyle \mathrm{CEA} = 2\pi \, \sigma _x \sigma _y \sqrt{1 - \rho ^2}$	Area of the $95\%$ bivariate normal ellipse
Mean distance (MD)	$\displaystyle \mathrm{MD} = \frac{1}{n} \sum _{i=1}^n \sqrt{(x_i - \bar{x})^2 + (y_i - \bar{y})^2}$	Mean radial distance of CoP from its mean position
Mean velocity (MV)	$\displaystyle \mathrm{MV} = \frac{1}{(n - 1)\,\Delta t} \sum _{i=1}^{n - 1} \sqrt{(x_{i+1} - x_i)^2 + (y_{i+1} - y_i)^2}$	Mean CoP speed across the trial
Standard deviation (SD), ML/AP	$\displaystyle \mathrm{SD}_{\mathrm{ML}} = \sqrt{\frac{1}{n} \sum _{i=1}^n (x_i - \bar{x})^2}$	Variability of CoP along each axis
	$$\displaystyle \mathrm{SD}_{\mathrm{AP}} = \sqrt{\frac{1}{n} \sum _{i=1}^n (y_i - \bar{y})^2}$$	
Root mean square (RMS) radius	$\displaystyle \mathrm{RMS} = \sqrt{\frac{1}{n} \sum _{i=1}^n \big [ (x_i - \bar{x})^2 + (y_i - \bar{y})^2 \big ]}$	RMS radial dispersion (2D SD of zero-meaned CoP)
Maximum sway range, ML/AP	$\displaystyle \mathrm{Range}_{\mathrm{ML}} = \max (x_i) - \min (x_i)$	Peak-to-peak CoP excursion along each axis
	$$\displaystyle \mathrm{Range}_{\mathrm{AP}} = \max (y_i) - \min (y_i)$$	
Mean frequency (MFREQ)	$\displaystyle \mathrm{MFREQ} = \frac{\mathrm{MV}}{4\sqrt{2}\,\mathrm{MD}}$	Proxy for characteristic sway frequency (ratio of speed to amplitude)

Here, ($x_i,y_i$) are CoP samples at time $t_i$, $\Delta t=t_{i+1}-t_i$, $n$ is the number of samples, $\bar{x}$ and $\bar{y}$ are sample means, and $\rho$ is the ML–AP correlation

**Table 2:** Center-of-pressure to center-of-mass (CoP–CoM) metrics used in this study

Metric	Formula	Description
Path length (PL)	$$\displaystyle \mathrm{PL} = \sum _{i=1}^{n - 1} \sqrt{ \begin{aligned}&\big [ (x_{\mathrm{CoP}, i+1} - x_{\mathrm{CoM}, i+1}) - (x_{\mathrm{CoP}, i} - x_{\mathrm{CoM}, i}) \big ]^2 \\&\quad + \big [ (y_{\mathrm{CoP}, i+1} - y_{\mathrm{CoM}, i+1}) - (y_{\mathrm{CoP}, i} - y_{\mathrm{CoM}, i}) \big ]^2 \end{aligned} }$$	Total distance traveled by CoP relative to CoM during the trial
Mean distance (MD)	$\displaystyle \mathrm{MD} = \frac{1}{n} \sum _{i=1}^n \sqrt{(x_{\mathrm{CoP}, i} - x_{\mathrm{CoM}, i})^2 + (y_{\mathrm{CoP}, i} - y_{\mathrm{CoM}, i})^2}$	Mean CoP–CoM separation; smaller values indicate greater stability
Root mean square (RMS) distance	$\displaystyle \mathrm{RMS} = \sqrt{\frac{1}{n} \sum _{i=1}^n \big [ (x_{\mathrm{CoP}, i} - x_{\mathrm{CoM}, i})^2 + (y_{\mathrm{CoP}, i} - y_{\mathrm{CoM}, i})^2 \big ]}$	Dispersion of CoP–CoM distances along both axes
Maximum sway range, ML/AP	$\displaystyle \mathrm{Range}_{\mathrm{ML}} = \max (x_{\mathrm{CoP}} - x_{\mathrm{CoM}}) - \min (x_{\mathrm{CoP}} - x_{\mathrm{CoM}})$	Peak-to-peak CoP–CoM deviations in ML and AP directions
	$$\displaystyle \mathrm{Range}_{\mathrm{AP}} = \max (y_{\mathrm{CoP}} - y_{\mathrm{CoM}}) - \min (y_{\mathrm{CoP}} - y_{\mathrm{CoM}})$$	
Correlation, ML/AP	$\displaystyle \mathrm{corr}(x_{\mathrm{CoP}},\, x_{\mathrm{CoM}}), \quad \mathrm{corr}(y_{\mathrm{CoP}},\, y_{\mathrm{CoM}})$	Linear association between CoP and CoM trajectories along each axis
Mean velocity (MV)	$$\displaystyle \mathrm{MV} = \frac{1}{(n - 1)\,\Delta t} \sum _{i=1}^{n - 1} \sqrt{ \begin{aligned}&\big [ (x_{\mathrm{CoP}, i+1} - x_{\mathrm{CoM}, i+1}) - (x_{\mathrm{CoP}, i} - x_{\mathrm{CoM}, i}) \big ]^2 \\&\quad + \big [ (y_{\mathrm{CoP}, i+1} - y_{\mathrm{CoM}, i+1}) - (y_{\mathrm{CoP}, i} - y_{\mathrm{CoM}, i}) \big ]^2 \end{aligned} }$$	Mean relative speed of CoP with respect to CoM
Phase lag, ML/AP	$\Delta t_{\mathrm{ML}},\; \Delta t_{\mathrm{AP}}$ from cross-correlation (positive lag indicates CoP leads CoM; negative lag indicates CoP lags CoM)	Temporal delay between CoP and CoM motions along ML and AP axes

Here, ($x_{\mathrm{CoP},i},\,y_{\mathrm{CoP},i}$) and ($x_{\mathrm{CoM},i},\,y_{\mathrm{CoM},i}$) denote paired CoP and CoM samples at time $t_i$, $\Delta t=t_{i+1}-t_i$, and $n$ the number of samples

#### Wobble Board (WB) Metrics

Our basic unit of analysis was the “roll cycle,” defined as the interval between two consecutive zero crossings of the wobble board’s angular velocity, measured relative to the horizontal plane. Zero crossings were identified by tracking the roll angular velocity over time—defined by rotation around the board’s sagittal axis—and detecting changes in its sign (from positive to negative or vice versa). To avoid ambiguity in sign determination, frames where the roll angle was exactly zero were offset by a small numerical value (e.g., $10^{-6}$). Unlike methods that define cycle boundaries using consecutive local maxima of the roll angle, we chose zero crossings because they represent a stable alignment of the board in a neutral horizontal position, analogous to the stable stance phases that delineate stride intervals in gait analysis [55]. Roll cycles provided discrete peak angular excursions and cycle durations analogous to stride intervals and stride lengths in walking. The resulting series of peak angular excursions and cycle durations enabled the examination of both linear and nonlinear properties of the wobble-board dynamics—WB metrics (Table 3). Furthermore, this approach facilitated the application of Detrended Moving-Average Analysis (DMA) to quantify the long-range temporal correlations in the cyclical motion of the body-board system, aligning with established methodologies in human gait and posture research (cf. [56–58]).

We computed the Hurst exponents to quantify long-range temporal correlations in two key aspects of wobble-board dynamics (Table 3): (*i*) Peak angular excursion ($H_{\mathrm {peak\;angular\;excursion}}$): This exponent characterizes the temporal persistence in the maximum tilt angles achieved during each oscillation cycle. Values were calculated from the series of peak angular excursions from the horizontal position. (*ii*) Cycle durations ($H_{\mathrm {cycle\;duration}}$): This exponent quantifies the temporal correlations in the intervals between successive cycles. The analysis was performed on the sequence of periods between consecutive peak excursions. Both Hurst exponents were computed using $2$nd order DMA, where $H\!\approx \!0.5$ indicates uncorrelated random behavior, $0.5\!<\!H\!<\!1.0$ suggests persistent long-range temporal correlations, and $0\!<\!H\!<\!0.5$ indicates anti-persistent behavior. The process of DMA has been detailed previously [39–41].

**Table 3:** Wobble-board (WB) metrics used in this study

Metric	Formula	Description
Mean peak angular excursion	$\displaystyle \bar{\theta }_{\mathrm{peak}} = \frac{1}{n} \sum _{i=1}^n \theta _{\mathrm{peak}, i}$	Mean cycle-to-cycle excursion amplitude (spatial magnitude)
RMS peak angular excursion	$\displaystyle \mathrm{RMS}_{\theta } = \sqrt{\frac{1}{n} \sum _{i=1}^n \theta _{\mathrm{peak},i}^2}$	Dispersion of excursion magnitudes across cycles
RMS angular velocity	$\displaystyle \mathrm{RMS}_{\dot{\theta }} = \sqrt{\frac{1}{N} \sum _{k=1}^{N} \dot{\theta } (t_k)^2}$ ($N$ is the number of time samples in the trial; $\dot{\theta }(t_k)$ is sampled at the motion-capture rate)	Overall variability of instantaneous board speed within a trial
Mean cycle duration	$\displaystyle \bar{T} = \frac{1}{n} \sum _{i=1}^n T_i$	Mean time per cycle (temporal rhythm)
RMS cycle duration	$\displaystyle \mathrm{RMS}_{T} = \sqrt{\frac{1}{n} \sum _{i=1}^n T_i^2}$	Dispersion of cycle timing across cycles
Number of cycles	$n=$ count of completed cycles (a cycle is defined between consecutive zero-crossings of roll angular velocity (sign change), yielding one left–right (or right–left) oscillation)	Total number of roll cycles in the trial
Cycle frequency	$\displaystyle f = \frac{1}{\bar{T}}$	Mean cycle rate (Hz), reciprocal of mean duration
Hurst exponent of peak angular excursions	$H_{\mathrm {peak\;angular\;excursion}}$ via $2$nd-order DMA	Long-range temporal dependence of the $\{\theta _{\mathrm{peak},i}\}$ series
Hurst exponent of cycle durations	$H_{\mathrm {cycle\;duration}}$ via $2$nd-order DMA	Long-range temporal dependence of the $\{T_i\}$ series

Here, $\theta _{\mathrm{peak},i}$ is the $i$th peak angular excursion ($^{\circ }$), $T_i$ the $i$th cycle duration (s), $\dot{\theta }_i$ the instantaneous angular velocity ($^{\circ }$/s), and $n$ the number of completed cycles in a trial

In brief, DMA proceeds by: (*i*) integrating the mean-removed series; (*ii*) subtracting a smooth trend estimated with an $m$th-order Savitzky–Golay filter (for second-order DMA we use even $m$ and an odd filter window) [59–62]; (*iii*) computing the root-mean-square fluctuation $F(s)$ of the detrended signal over windows of size $s$; and (*iv*) estimating the scaling exponent $\alpha$ by linear regression of $\log F(s)$ versus $\log s$. Because our inputs are difference-type series, we treat them as fractional Gaussian noise and report the Hurst exponent as $H\!=\!\alpha$. For second-order DMA we apply the standard timescale correction $\tilde{s}\!=\!s/1.93$ to maintain time–frequency alignment [63]. By convention, $H\!\approx \!0.5$ denotes uncorrelated variability, $H\!>\!0.5$ indicates persistent long-range dependence, and $H\!<\!0.5$ indicates anti-persistence. To confirm that structure exceeded finite-sample randomness, we also computed $H$ for phase-shuffled surrogates of each series and compared them with the originals.

### Statistical Analysis

We used linear mixed-effects models to examine the influence of task condition (“TMT” vs. no TMT) and trial (“T2” for trial 2 vs. trial 1) on TMT score and the first seven of the nine WB metrics—mean peak angular excursion, RMS peak angular excursion, RMS angular velocity, mean cycle duration, RMS cycle duration, number of cycles, and cycle frequency. We used another linear mixed-effects model to examine the influence of task condition (“TMT” vs. no TMT) variable (“peakAngularExcursion” vs. cycleDuration), type (“Original” vs. Shuffled), and trial (“T2” for trial 2 vs. trial 1) on the Hurst exponent, $H$, obtained using $2$nd-order DMA. In each of these models, we included the random factor of participant identity by allowing the intercept to vary across participants. We performed all regression analyses in R [64] using the function lmer() from the package lme4 [65] and set the threshold for statistical significance at alpha level $0.05$ using package lmerTest [66]. Linear mixed-effects models yielded coefficients $\beta$ for each covariate, representing the average change in the dependent variable. We present estimated $\beta$ from the linear mixed-effects model and corresponding $p$-value.

We tested whether cyclical wobble-board dynamics would exhibit person-specific “signatures” of balance control, and we computed intraclass correlation coefficients (ICCs) for each WB metric to assess the reliability of repeated measurements within individuals. Specifically, a two-way mixed-effects model with absolute agreement $[\mathrm{ICC}(3,\,1)]$ was used to quantify the stability of repeated measurements relative to overall variability. We report ICC values and their $95\%$ confidence intervals (CIs) to estimate the reliability assessment’s precision. The ICC was computed using the icc() function from the irr package [67] in R [64]. The data were first reshaped into a wide format to accommodate repeated measures across participants and conditions.

We employed a multivariate analysis approach to evaluate the relationships and potential redundancy between different types of balance metrics. First, all metrics were standardized using $z$-score normalization to account for different measurement scales. Principal Component Analysis (PCA) [68, 69] was performed on the complete dataset comprising $28$ balance metrics ($11$ CoP metrics, $10$ CoP–CoM metrics, and $7$ WB metrics) collected from $29$ participants, each performing two trials under two conditions (total observations $=\!116$). The PCA transformation was conducted using the covariance matrix of the standardized data. The number of principal components was determined by the rank of the data matrix, yielding $28$ orthogonal components. The relative contribution of each metric type (CoP, CoP–CoM, WB) to each principal component was calculated as the sum of squared loadings for that metric category, normalized by the total variance explained by the component. To specifically examine the relationship between whole-body metrics and the other measurement approaches, two separate Canonical Correlation Analyses (CCA) were performed: one between WB metrics and CoP metrics and another between WB metrics and CoP–CoM metrics. CCA identifies linear combinations of variables in each set (canonical variates) that maximize the correlation between sets. The number of canonical correlations was limited by the smaller dimension of each pair of metric sets (in this case, four, determined by the number of WB metrics). Trial-to-trial consistency was assessed by calculating Euclidean distances between repeated trials in the PC1–PC2 space for each participant and condition. These distances measure within-subject variability, with smaller distances indicating better reproducibility between trials. Between-condition differences were visualized using the first two principal components, which typically capture the majority of the variance in the data. The analysis was implemented in MATLAB using built-in functions for PCA (pca()) and CCA (canoncorr()).

## Results

### Trail Making Task (TMT) Performance

There was no significant difference in TMT score between the two support conditions (mean ± s.d. = 45.7 ± 12.4 in the “wobble board, no TMT” condition, WBNT, and 43.8 ± 9.7 in the “wobble board, TMT” condition, WBWT; $\beta \!=\!-\,1.86,\ t\!=\!-\,1.33,\ p\!=\!0.187$). This absence of a dual-task effect on TMT performance suggests that participants were able to maintain cognitive performance while balancing on the wobble board, despite the increased postural demands. Together with the unchanged Hurst exponents for postural sway, this finding indicates that the postural control system effectively accommodated concurrent cognitive load without compromising task execution. Such stability under combined demands implies a degree of robustness or adaptive resource allocation within the posture–cognition system, consistent with theories proposing flexible prioritization of postural stability when attentional resources are shared. The results therefore reinforce the interpretation that the proactive–emergent organization of dynamic balance is preserved under moderate cognitive challenge, supporting accounts of load-resilient coordination dynamics during unstable stance.

### Distinct Temporal Patterns in Proactive and Emergent Balance Control

**Fig. 3:**
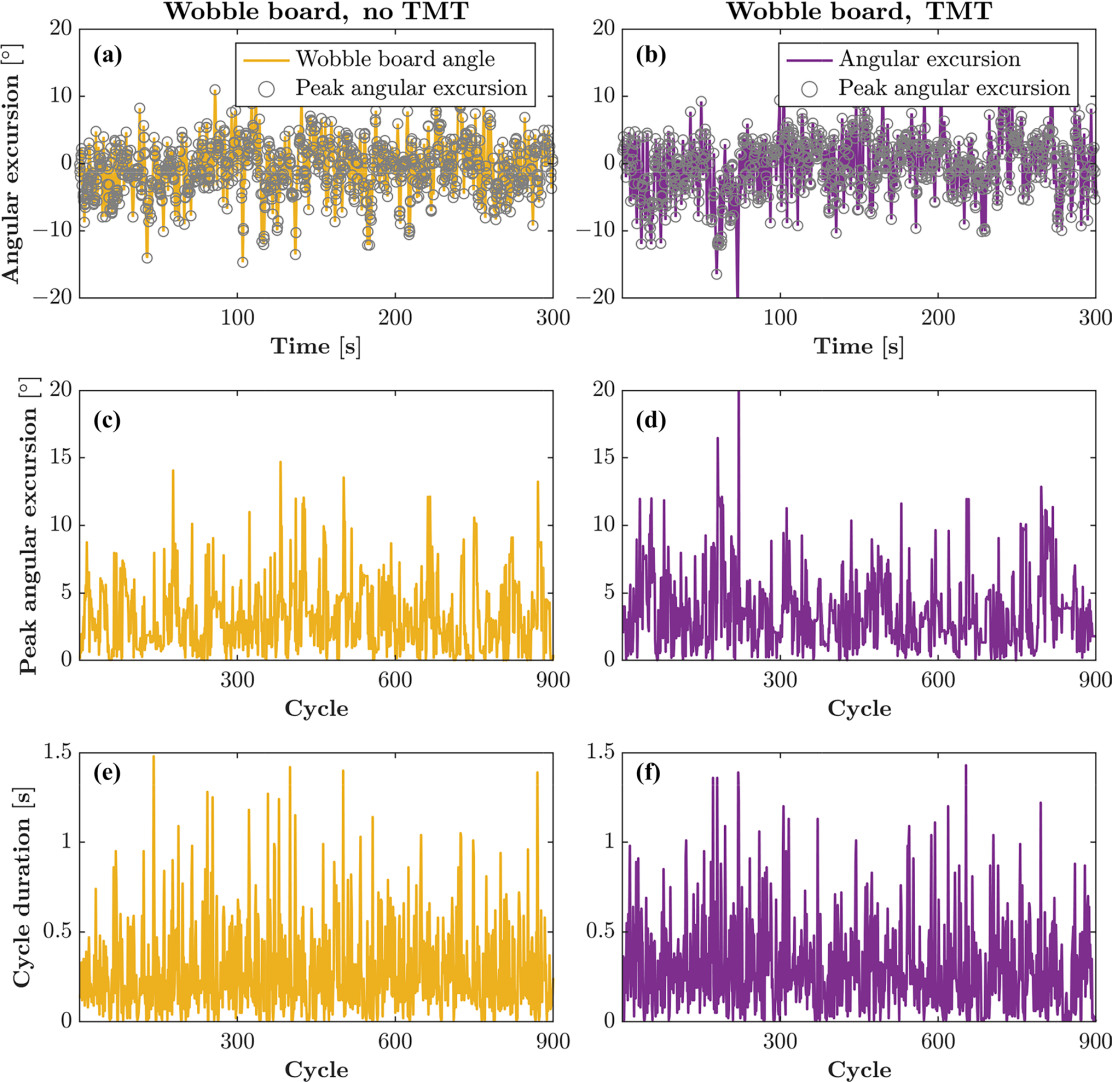
Wobble-board dynamics with and without a concurrent Trail Making Task (TMT) for a representative participant. Panels **a** and **b** show the time series of wobble-board angles (solid lines) as well as peak angular excursions (circles) for the wobble board, no TMT (WBNT) and wobble board, TMT (WBWT) conditions, respectively. Panels **c** and **d** depict the peak angular excursions over consecutive cycles for the two conditions. Panels **e** and **f** display the corresponding series of cycle durations

The results detail wobble-board dynamics under the two experimental conditions: wobble board, no TMT (WBNT) and wobble board, TMT (WBWT). Fig. 3 first shows, for a representative participant, the time series of wobble-board angle relative to horizontal with detected peak angular excursions for WBNT (Fig. 3a) and WBWT (Fig. 3b). It then presents the peak angular excursions across consecutive cycles for the same two conditions (Fig. 3c, d), followed by the corresponding cycle-duration series (Fig. 3e, f).

**Fig. 4:**
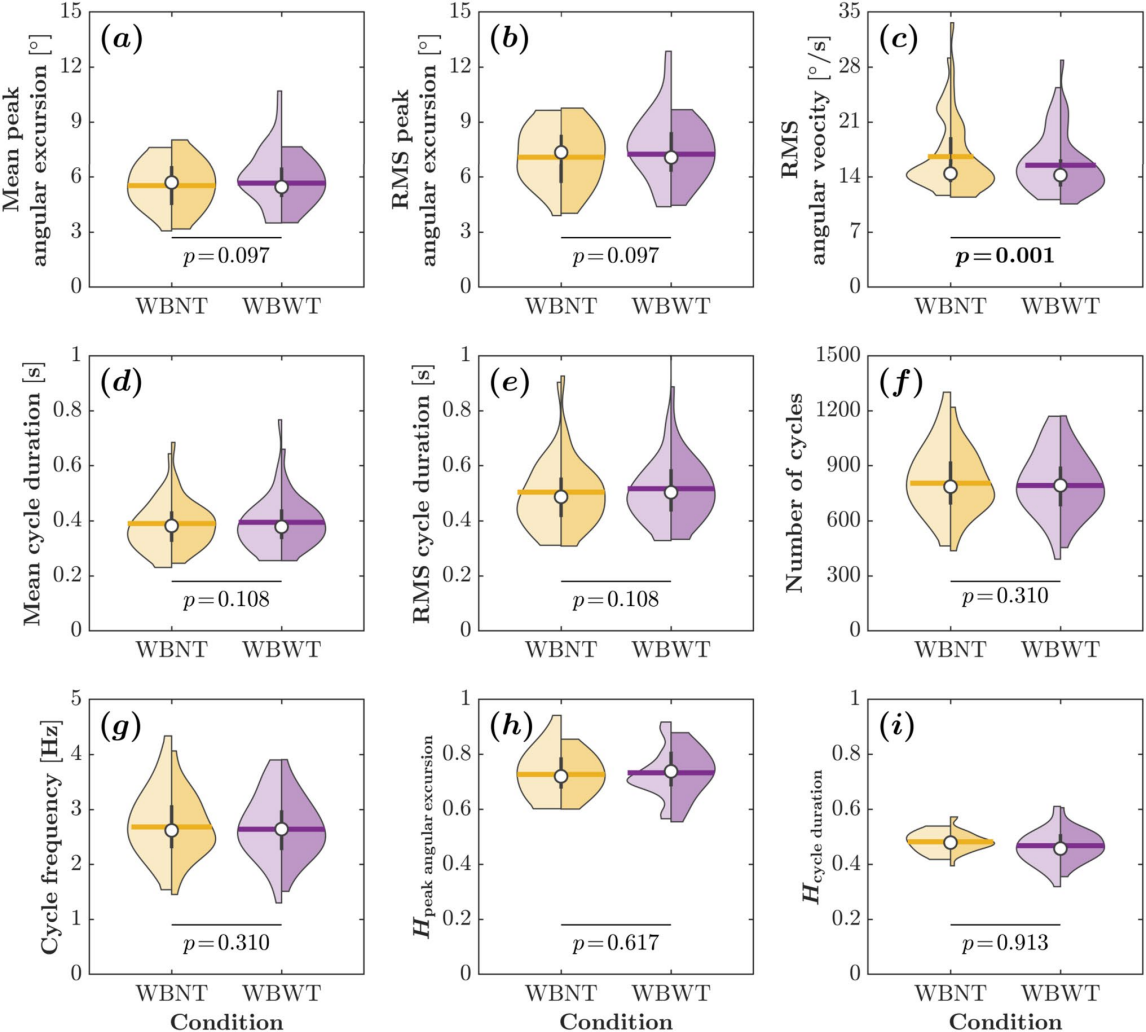
Comparison of various wobble-board metrics between WBNT (wobble board, no TMT) and WBWT (wobble board, TMT) conditions. Each panel depicts a different metric: **a** mean peak angular excursion, **b** RMS peak angular excursion, **c** RMS angular velocity, **d** mean cycle duration, **e** RMS cycle duration, **f** number of cycles, **g** cycle frequency, **h** Hurst exponent of peak angular excursions ($H_{\mathrm {peak\;angular\;excursion}}$), and **i** Hurst exponent of cycle durations ($H_{\mathrm {cycle\;duration}}$). The $p$-values for comparisons between no-TMT and TMT conditions are indicated beneath the violin plots, showing no significant differences $(p\!>\!0.05)$ across all metrics except for RMS angular velocity. Each violin plot’s left and right half depict data distributions for the first and second trials. Horizontal bars indicate mean, white circles indicate median, and vertical bars indicate interquartile ranges for trial 2 (25th and 75th percentiles; $n\!=\!29$ participants)

Across all $58$ trials ($2$ trials for each of $29$ participants), the metrics are reported in the same order as Fig. 4. The mean peak angular excursion was mean ± s.d. = 5.59 $\pm \,1.23^{\circ }$ in WBNT and $5.89\!\pm \!1.44^{\circ }$ in WBWT (Fig. 4a). Variability measures for excursion amplitude and speed—RMS peak angular excursion and RMS angular velocity—are shown in Fig. 4b and c, respectively. The mean cycle duration was $0.38\pm 0.09$ s in WBNT and $0.39\pm 0.10$ s in WBWT (Fig. 4d), and the RMS cycle duration appears in e. The number of cycles was $823\pm 183$ in WBNT and $802\pm 171$ in WBWT (Fig. 4f), and the cycle frequency was $2.74\pm 0.61$ Hz in WBNT and $2.67\pm 0.57$ Hz in WBWT (Fig. 4g). Together, these panel-aligned summaries provide a coherent overview of the wobble-board oscillations across conditions and set the stage for subsequent analyses of variability and long-range temporal organization.

**Fig. 5:**
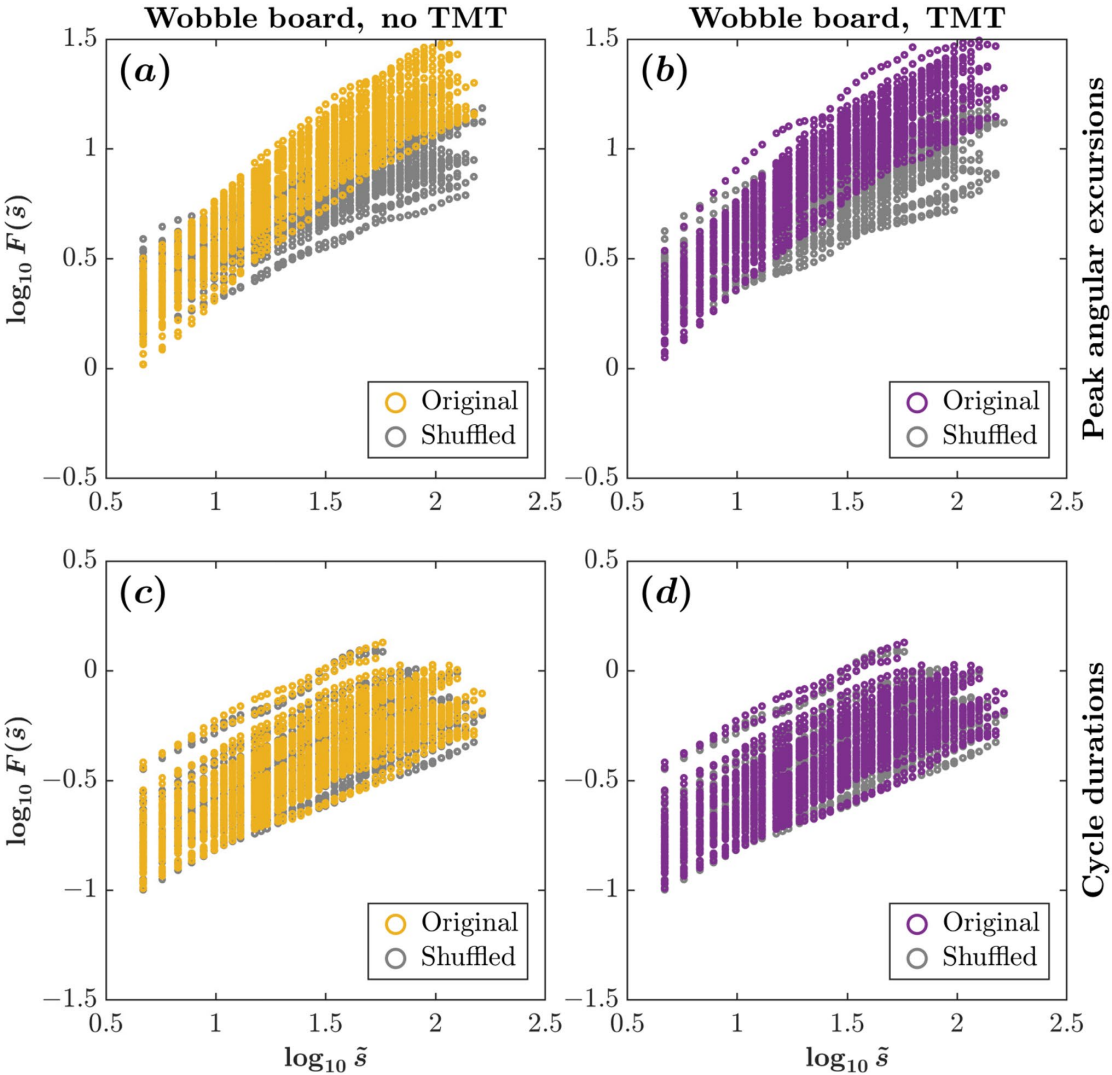
Fluctuation functions, $\boldsymbol{F}(\tilde{\boldsymbol{s}})$, plotted against scale, $\tilde{\boldsymbol{s}}$, on a log–log scale. Panels **a** and **b** correspond to peak angular excursions, while panels **c** and **d** represent cycle durations. Results are shown for trials without Trail Making Task (TMT) (left panels: **a** and **c**) and with TMT (right panels: **b** and **d**). Original series data (colored circles) are compared against shuffled surrogates (gray circles) to assess long-range temporal correlations. Yellow and purple points denote original data for no-TMT and TMT conditions, respectively, with systematic deviations from shuffled data indicating long-range temporal correlations in peak angular excursions but not in cycle durations ($n\!=\!58$; $29$ participants, two trials/condition)

The analysis of temporal structure in the wobble-board spatiotemporal dynamics—peak angular excursion series versus cycle duration series—revealed a clear dissociation between actively regulated and emergent variables. The Hurst exponent $H$, which indexes long-range temporal dependence, was markedly higher for the peak-angular-excursion series (WBNT: $0.73\pm 0.08$; WBWT: $0.73\pm 0.09$; Figs. 4h and 5a, b) than for the cycle-duration series (WBNT: $0.48\pm 0.04$; WBWT: $0.46\pm 0.06$; Figs. 4i and 5c, d). This pattern supports the view that feedback-based control actively constrains spatial excursions—selectively reversing roll direction once instability or excessive tilt approaches a threshold—while the near-random timing of cycles reflects dynamics that emerge passively from board–body mechanics. In short, proactive control prioritizes limiting large spatial deviations that threaten stability, whereas temporal cadence remains flexible and largely unconstrained, allowing adaptation to changing task demands.

We further tested for temporal randomness by calculating the Hurst exponent ($H$) on phase-shuffled versions of the peak angular excursion and cycle duration series. As expected for random signals, $H$ was approximately $0.5$ for both variables (peak angular excursion: WBNT: $0.46\!\pm \!0.04$, WBWT: $0.46\!\pm \!0.04$; cycle duration: WBNT: $0.46\!\pm \!0.04$, WBWT: $0.46\!\pm \!0.04$; grey scatters in Fig. 5), indicating fluctuations devoid of long-range temporal correlations. Linear mixed-effects modeling corroborated these findings: the original (non-shuffled) series showed a positive effect ($\beta \!=\!2.04\!\times \!10^{-2},\ t\!=\!2.11,\ p\!=\!0.035$; Table 4), demonstrating that temporal structure in the observed data differs significantly from randomness. Moreover, the interaction between dynamic variable and series type was large and robust ($\beta \!=\!2.43\!\times \!10^{-1},\ t\!=\!17.78,\ p\!<\!2.220\!\times \!10^{-16}$; Table 4), confirming that peak angular excursions—unlike cycle durations—exhibit pronounced long-range organization consistent with feedback-based control. These results suggest that the central nervous system actively regulates spatial excursions at peaks to maintain balance, whereas cycle timing remains near-random and emergent.

**Table 4:** Outcomes of the linear mixed-effects modeling$^{1}$ examining the influence of task condition (“TMT” vs. no TMT) variable (“peakAngularExcursion” vs. cycleDuration), type (“Original” vs. Shuffled), and trial (“T2” for trial 2 vs. trial 1) on the Hurst exponent, $H$, obtained using $2$nd-order DMA

$\mathrm{Factor}$	$\beta \!\pm \!s.e.$	$t$	$p^{2}$
(Intercept)	$4.61\!\times \!10^{-1}\!\pm \!7.77\!\times \!10^{-3}$	$59.30$	$\boldsymbol{2.220\!\times \!10^{-16}}$
TMT	$-\,8.83\!\times \!10^{-4}\!\pm \!9.63\!\times \!10^{-3}$	$-\,0.09$	$0.927$
peakAngularExcursion	$1.34\!\times \!10^{-3}\!\pm \!9.63\!\times \!10^{-3}$	$0.14$	$0.889$
Original	$2.04\!\times \!10^{-2}\!\pm \!9.63\!\times \!10^{-3}$	$2.11$	$\boldsymbol{0.035}$
trialT2	$3.03\!\times \!10^{-3}\!\pm \!4.83\!\times \!10^{-3}$	$0.63$	$0.531$
TMT:peakAngularExcursion	$-\,4.50\!\times \!10^{-3}\!\pm \!1.36\!\times \!10^{-2}$	$-\,0.33$	$0.741$
TMT:Original	$-\,1.73\!\times \!10^{-2}\!\pm \!1.36\!\times \!10^{-2}$	$-\,1.27$	$0.205$
peakAngularExcursion:Original	$2.43\!\times \!10^{-1}\!\pm \!1.37\!\times \!10^{-2}\!$	$17.78\!$	$\boldsymbol{2.220\!\times \!10^{-16}}$
TMT:peakAngularExcursion:Original	$2.21\!\times \!10^{-2}\!\pm 1.93\!\times \!10^{-2}$	$1.14$	$0.254$

$$^{1}H \sim (\mathrm{taskCondition} * \mathrm{variable} * \mathrm{type}) + \mathrm{trial} + (1 | \mathrm{participant})$$

^2^Boldfaced values indicate statistical significance at $p < 0.05$

### Impact of Concurrent Task Interference on Wobble-Board Dynamics

We used linear mixed-effects models to test the impact of the concurrent TMT on the spatiotemporal dynamics of wobble-board control across all WB metrics. Task condition (WBNT vs. WBWT) was included as a fixed effect for every model; for the two Hurst-exponent outcomes, *type* (original vs. shuffled) was additionally included. The mean peak angular excursion, representing the maximum tilt of the wobble board during each cycle, was unaffected by the concurrent task ($\beta \!=\!0.15^\circ ,\ t\!=\!1.68,\ p\!=\!0.097$; Fig. 4a). The same held for RMS peak angular excursion ($\beta \!=\!0.15^\circ ,\ t\!=\!1.68,\ p\!=\!0.097$; Fig. 4b). By contrast, RMS angular velocity was significantly smaller during the TMT ($\beta \!=\!-\,0.92^\circ /\text {s},\ t\!=\!-\,3.30,\ p\!=\!0.001$; Fig. 4c). Temporal metrics showed no reliable task effects: mean cycle duration ($\beta \!=\!-\,0.01\,\text {s},\ t\!=\!1.63,\ p\!=\!0.108$; Fig. 4d), RMS cycle duration ($\beta \!=\!0.01\,\text {s},\ t\!=\!-\,1.63,\ p\!=\!0.108$; Fig. 4e), number of cycles ($\beta \!=\!-\,13.31,\ t\!=\!-\,1.02,\ p\!=\!0.310$; Fig. 4f), and cycle frequency ($\beta \!=\!-\,0.04\,\text {Hz},\ t\!=\!-\,1.02,\ p\!=\!0.310$; Fig. 4g) did not differ between WBNT and WBWT. Thus, participants maintained a consistent cadence across single- and dual-task conditions. Together, these results indicate that the concurrent task modestly reduced angular speed (smaller RMS angular velocity) without altering the amplitude or timing of wobble-board oscillations. In other words, cognitive load from the TMT did not disrupt the fundamental spatiotemporal organization of balance control.

The Hurst exponent for the peak angular excursion, indicative of persistent control strategies, remained consistent across the two task conditions. The model revealed no significant main effect of the concurrent TMT on the $H$-values ($\beta \!=\!-\,0.01,\ t\!=\!-\,0.50,\ p\!=\!0.617$; Fig. 4h), suggesting that the dual-task condition did not disrupt the active regulation of board excursions. Thus, it appears that the nervous system maintains proactive control of wobble-board amplitude regardless of cognitive task demands. For the cycle duration, characterized by random, uncorrelated patterns, the analysis indicated no significant effect of the concurrent TMT on the $H$-values ($\beta \!=\!-\,0.01,\ t\!=\!-\,0.11,\ p\!=\!0.913$; Fig. 4i). The mean $H$-values for the cycle duration consistently approximated $0.5$ in both single- and concurrent task conditions, reflecting stochastic dynamics that remained unaffected by the addition of a task demanding cognitive engagement.

### Consistency and Variability in Wobble-Board Dynamics Across Conditions and Trials

**Fig. 6:**
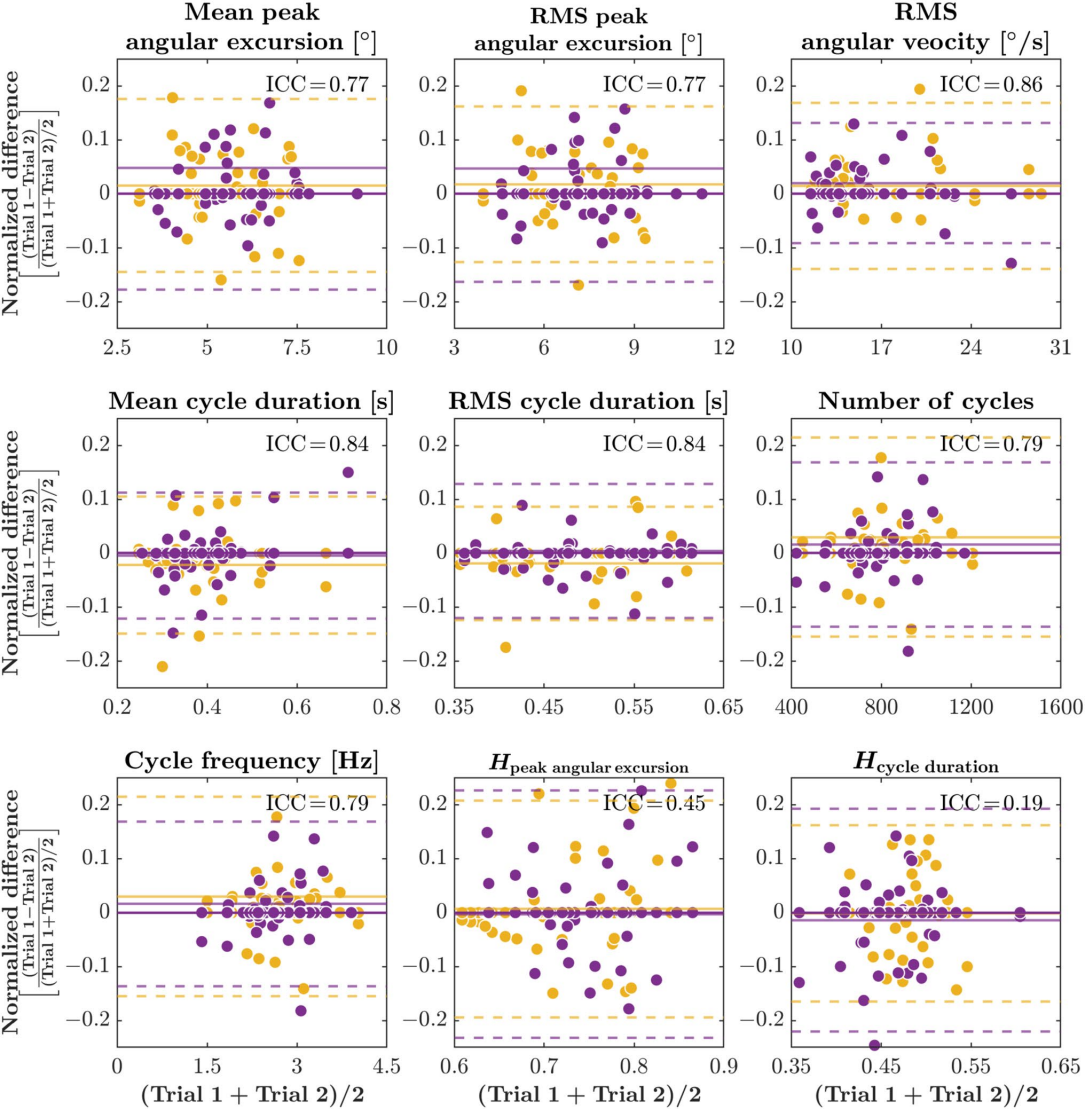
Bland–Altman plots illustrating test-retest agreement across nine wobble-board metrics. The difference between Trial 1 and Trial 2 is plotted against the average for each participant. Each panel depicts a different metric: **a** mean peak angular excursion, **b** RMS peak angular excursion, **c** RMS angular velocity, **d** mean cycle duration, **e** RMS cycle duration, **f** number of cycles, **g** cycle frequency, **h** Hurst exponent of peak angular excursions ($H_{\mathrm {peak\;angular\;excursion}}$), and **i** Hurst exponent of cycle durations ($H_{\mathrm {cycle\;duration}}$). Solid horizontal lines indicate the mean difference, while dashed lines denote the limits of agreement ($\pm \!1.96\,s.d.$). Yellow and purple circles correspond to WBNT and WBWT conditions. Measures with tighter clustering around zero (e.g., panels **a–g**) suggest higher test–retest reliability, while those with broader scatter (e.g., panels **h, i**) indicate lower reliability. $n\!=\!29$ participants.

We hypothesized that the cyclical wobble-board dynamics would exhibit person-specific “signatures” of balance control and thus remain consistent across repeated trials and the no-TMT and TMT conditions. By contrast, more fractal-like metrics quantifying the long-range temporal correlations in the wobble-board dynamics might capture trial-to-trial fluctuations. To test this hypothesis, we computed intraclass correlation coefficients (ICCs) using a two-way mixed-effects model with absolute agreement $[\mathrm{ICC}(3,\,1)]$, which quantifies the stability of repeated measurements within individuals relative to overall variability. As illustrated in the Bland–Altman plots, the first seven metrics demonstrated moderate-to-high ICC values—mean peak angular excursion ($0.77,\ 95\%\ \mathrm{CI}\,[0.64,\,0.87]$), RMS peak angular excursion ($0.77,\ 95\%\ \mathrm{CI}\,[0.64,\,0.87]$), RMS angular velocity ($0.86,\ 95\%\ \mathrm{CI}\,[0.77,\,0.92]$); mean cycle duration ($0.84,\ 95\%\ \mathrm{CI}\,[0.75,\,0.91]$), RMS cycle duration ($0.84,\ 95\%\ \mathrm{CI}\,[0.75,\,0.91]$), number of cycles ($0.79,\ 95\%\ \mathrm{CI}\,[0.67,\,0.88]$), cycle frequency ($0.79,\ 95\%\ \mathrm{CI}\,[0.67,\,0.88]$), Fig. 6a–g). These patterns indicate that these cyclical features of wobble-board dynamics reflect consistent individual control strategies, with each participant tending to produce similar dynamics across sessions. In contrast, the Hurst exponents quantifying the long-range temporal correlations in peak angular excursion series ($H_{\mathrm {peak\;angular\;excursion}}$) and cycle duration series ($H_{\mathrm {cycle\;duration}}$) showed much lower ICCs ($0.45,\ 95\%\ \mathrm{CI}\,[0.26,\,0.64]$ and $0.19,\ 95\%\ \mathrm{CI}\,[0.02,\,0.40]$, respectively; Fig. 6h, i), suggesting that these fractal-like properties of the wobble-board dynamics are more sensitive to moment-to-moment variability across trials and conditions. Thus, whereas the cyclical metrics indexed stable, person-specific control signatures, the Hurst-exponent measures showed poor within-person reliability, reflecting greater sensitivity to trial- and condition-level fluctuations.

### Relationships Between Wobble-Board Dynamics and Traditional CoP and CoP–CoM Metrics

**Fig. 7:**
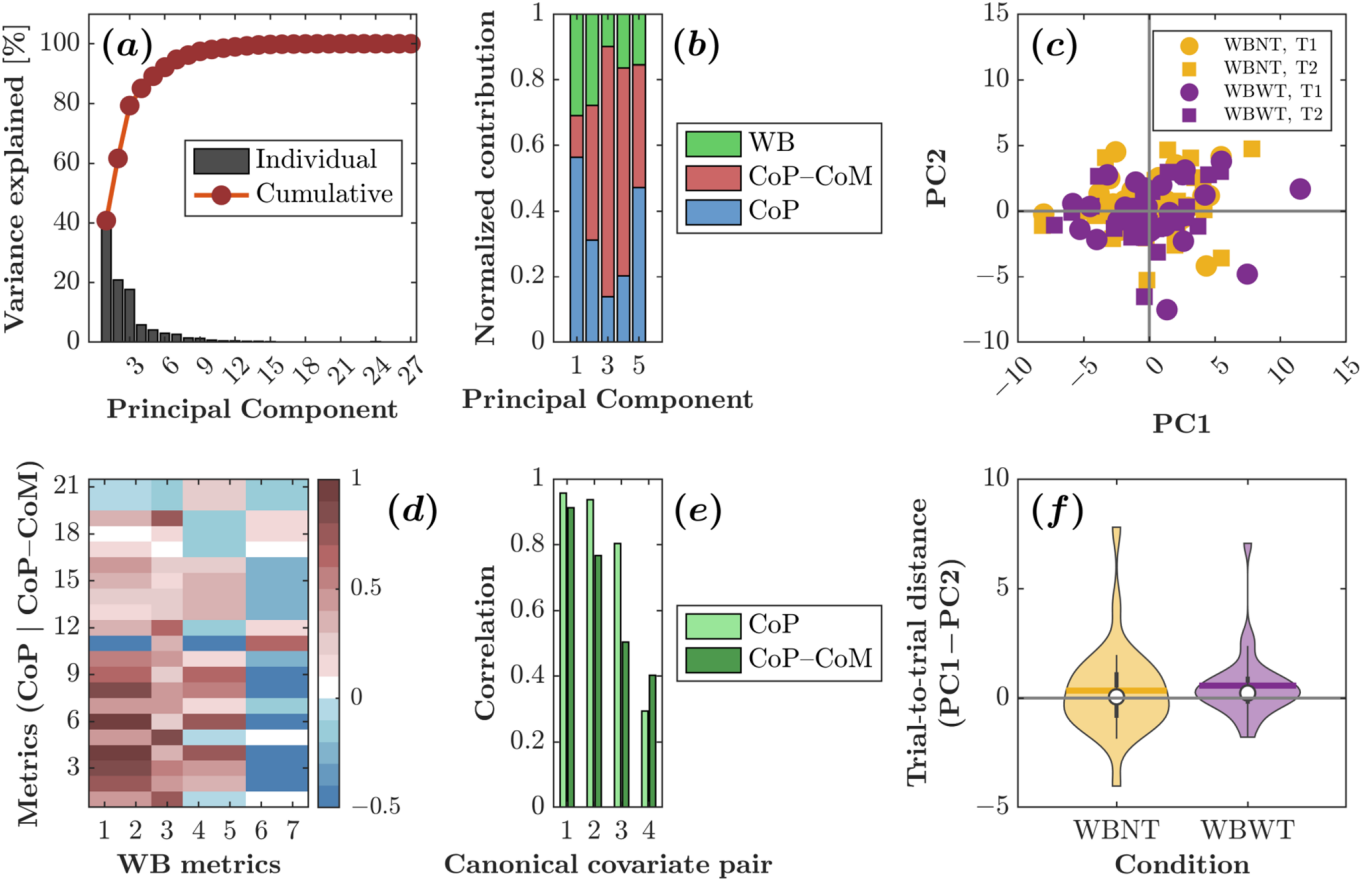
Principal Component Analysis (PCA) of the 28 balance metrics (11 CoP, 10 CoP–CoM, and 7 WB metrics) collected from 29 participants performing two trials under two conditions revealed strong dimensional reduction potential. **a** The first three principal components account for approximately $80\%$ of the total variance, with PC1 alone explaining about $40\%$. **b** Contributions of different metric types to the principal components highlighting CoP metrics as dominant in PC1 ($>\!55\%$), with CoP–CoM metrics dominating PC2 and PC3. **c** The PC1–PC2 score-plot showing considerable overlap between conditions and trials, but some outlier trials were evident, particularly in the WBWT condition. **d** A correlation heatmap between WB and other metrics revealing strong associations (dark red) for certain CoP–WB metric pairs and weak or negative correlations (blue) for others, suggesting redundancy and complementarity in metrics. **e** Canonical Correlation Analysis (CCA) demonstrating significant relationships between WB metrics and the two traditional metric sets, with two strong canonical correlations (approximately 0.9–0.95 and 0.75–0.95), indicating multidimensional shared information. **f** Trial-consistency analysis revealing slightly higher variability in WBNT than WBWT, with similar median trial-to-trial distances but notable outliers in both conditions, underscoring the importance of accounting for individual variability in balance assessments

We performed Principal Component Analysis (PCA) on $28$ balance metrics ($11$ CoP, $10$ CoP–CoM, $7$ WB) from $29$ participants (two trials under two conditions). PCA revealed substantial dimensionality reduction: the first three components explained $79.32\%$ of the variance, with PC1 alone accounting for $40.82\%$ (Fig. 7a). Metric-class loadings showed that CoP variables dominated PC1 ($56.39\%$), with smaller contributions from the other sets ($30.89\%$ for the WB metrics); in PC2 and PC3, contributions shifted, with CoP–CoM metrics loading more heavily (Fig. 7b). These patterns indicate complementary information across metric classes and suggest that the WB metrics occupy a distinct subspace captured more prominently in higher-order components.

The PC1–PC2 score-plot showed substantial overlap across conditions and trials (Fig. 7c), indicating that these components do not primarily encode trial- or condition-specific variance. A few outlier trials—more frequent in WBWT—suggest participant-specific strategies or challenges. The roughly uniform spread around the origin implies that PC1–PC2 capture natural inter-individual variability rather than systematic condition effects. The correlation heatmap (Fig. 7d) revealed strong pairwise associations between certain CoP variables (rows 1–11) and specific WB metrics (columns 1 & 2), indicating some redundancy, alongside weak or negative correlations that point to complementary information. Notably, high CoP–WB correlations were more prominent than CoP–CoM–WB correlations, suggesting CoP features may be more diagnostic of wobble-board dynamics than CoP–CoM measures. Canonical Correlation Analysis (CCA) corroborated substantial shared structure between WB and the traditional metric sets, with the first two canonical correlations of 91.25–95.78 and 76.76–93.76 (Fig. 7e); the presence of multiple significant canonical variates indicates multidimensional relationships that are not reducible to a single linear combination. Trial-consistency analysis showed slightly greater dispersion in WBNT than WBWT (Fig. 7f), with similar median within-subject distances across conditions, implying comparable reliability. Outliers in both conditions align with the score-plot pattern and may flag individuals with higher trial-to-trial variability—useful for personalized assessment and tracking during wobble-board–based interventions.

Taken together, these findings support the wobble board as a valid balance assessment instrument and highlight avenues for optimizing measurement. Strong canonical correlations with established CoP and CoP–CoM metrics indicate convergent validity, while condition-specific outliers and between-trial dispersion—especially in WBWT—underscore sensitivity to individual control strategies and challenges. This combination of convergent validity and person-level sensitivity positions wobble-board assessment as a promising platform for personalized evaluation. In practice, protocols can be tailored to individual needs while delivering clinically relevant information comparable to traditional methods.

## Discussion

The present investigation of balance control during wobble-board instability reveals fundamental insights into how the central nervous system manages unstable surface dynamics through a sophisticated interplay between spatial and temporal aspects of postural control. The distinct Hurst exponents for peak angular excursion ($H\!\approx \!0.75$) and cycle duration ($H\!\approx \!0.5$) highlight the nervous system’s use of specialized strategies to maintain balance on an unstable surface. While the Hurst exponent of $0.75$ for peak angular excursion magnitudes indicates persistent, proactive regulation, potentially through ankle proprioceptive feedback and reflex mechanisms, the Hurst exponent of $0.5$ for cycle duration reflects random, uncorrelated timings akin to Brownian motion. This differentiation in control dynamics suggests an elegant solution to the challenge of dynamic balance on wobble boards, where the nervous system actively regulates the amplitude of board movements while allowing the temporal organization or cadence of corrections to emerge naturally from the system’s dynamics. Such an arrangement appears to enhance control efficiency by focusing neural resources on task-critical aspects of the wobble-board dynamics while exploiting the natural mechanics of the board–body system for temporal coordination.

The mechanical coupling of movement amplitude and timing in postural control presents an intriguing scientific paradox: despite their physical interdependence, these parameters exhibit distinct neural control mechanisms. Our findings reveal that the nervous system preferentially regulates movement amplitude, allowing temporal patterns to emerge from system dynamics. This organizational principle challenges the traditional assumption that mechanically coupled parameters necessitate similar neural control strategies. Analysis of the underlying mechanics reveals why this control architecture is advantageous. By allocating neural resources specifically to amplitude control—the parameter most directly linked to task success—the nervous system achieves efficient control while leveraging passive dynamics for temporal coordination. The observed control architecture aligns with the principles of dynamic systems theory, where complex behaviors can emerge from the interaction between controlled and passive elements. In this framework, cycle duration emerges as a natural consequence of the system’s mechanical properties and constraints, eliminating the need for explicit temporal control [70–76]. These findings directly inform rehabilitation science—intervention protocols targeting cyclical behaviors should prioritize interventions targeting spatial control mechanisms while allowing temporal aspects to self-organize through natural dynamics. This approach promises more efficient rehabilitation strategies by focusing on parameters under active neural control rather than attempting to artificially modify emergent properties of the movement system.

The postural demands of wobble-board balancing appear to dominate suprapostural task performance, highlighting a hierarchical control architecture. Introducing a concurrent TMT did not alter the Hurst exponents for either excursion magnitude or cycle duration, implying that the proactive–emergent control organization of dynamic balance remained intact under cognitive load. Theoretically, this supports accounts in which maintaining stability on an unstable surface takes precedence over secondary tasks, consistent with ecological theories of postural control [77–79] and with the foundational role of postural stability in action [80, 81]. Clinically, this robustness could make wobble-board training attractive for contexts that combine balance with functional cognition; however, prior work documents dual-task interference in many settings [82–88], often amplified in older adults [89, 90] and neurological cohorts [91–95]. We therefore tested whether any trade-off manifested as decrements in TMT performance.

Participants did not exhibit a decrement in TMT performance while balancing on the wobble board, indicating no measurable dual-task cost on the suprapostural task at the difficulty tested—despite extensive evidence that posture–cognition dual-tasking can elicit interference under many conditions [82–88]. Taken with the unchanged Hurst exponents, this joint null suggests that the posture–cognition system accommodated concurrent load without detectable compromise to either stream. This pattern is consistent with (*i*) partial functional segregation of cognitive and postural control, (*ii*) sufficient spare capacity and/or flexible compensation that preserves both TMT accuracy/latency and the proactive–emergent architecture of balance, or (*iii*) an under-challenging suprapostural task (e.g., practice effects) that failed to tax shared resources. Conservatively, our data imply that the fractal-like balance metrics are load-resilient under the present demands and that concurrent light-to-moderate cognition can be performed without cognitive decrement during unstable stance. Notwithstanding, we do not rule out dual-task interference under heavier cognitive loads or in older and neurological cohorts, where resource limits may be tighter [82–88].

The preservation of wobble-board dynamics—particularly the *dispersion* of peak angular excursion and cycle duration—is consistent with muscle-spindle–mediated postural control. Fast, largely subcortical spindle feedback loops autonomously regulate muscle length and velocity [96–99], helping insulate core balance processes from concurrent cognitive demand. The dissociation in Hurst exponents further clarifies this hierarchy: persistent structure in peak angular excursion likely reflects continuous modulation of spindle gain via $\gamma$-motor drive to stabilize movement magnitude [100, 101], whereas the near-random timing of cycle duration is consistent with resonance emerging from spindle–muscle mechanics and board–body dynamics, requiring minimal active supervision [70–76]. In this view, the nervous system exploits intrinsic spindle–mechanical coupling to maintain spatial control while reserving higher-level resources for task-critical adjustments, yielding a load-resilient architecture under dual-task conditions.

The PCA results provide compelling evidence that variability in CoP and CoP–CoM metrics does not fully translate to functional performance in dynamic tasks like balancing on wobble boards. While CoP and CoP–CoM metrics capture variability in postural control [13, 14], they primarily reflect multiple static or quasi-static balance strategies, which are not directly indicative of the continuous, dynamic adjustments required for tasks involving wobble-board instability. WB metrics, by contrast, provide unique, task-specific information, as evidenced by their distinct contributions to higher-order principal components. CoP and CoP–CoM metrics dominated the first two principal components (PC1 and PC2), explaining the majority of the variance in the dataset. However, the information captured in these components reflects balance strategies that vary between individuals—for example, minimal sway versus controlled, larger sway—and may not be directly linked to performance in dynamic tasks. While contributing less to PC1 and PC2, the WB metrics dominated higher-order PCs, which capture subtle, complex dynamics like cyclical adjustments and oscillatory patterns inherent to dynamic balance control. Metrics like average cycle duration and cycle frequency exemplify the unique information provided by wobble-board metrics, which CoP and CoP–CoM metrics fail to represent.

The clinical significance of this distinction is substantial. Traditional CoP and CoP–CoM metrics, while valuable for research and basic assessment, may not fully capture the dynamic balance capabilities required for daily activities. By reflecting the continuous adjustments needed to maintain balance on an unstable surface, wobble-board dynamics might provide more functionally relevant information about a patient’s balance control. This is particularly important when considering activities that require dynamic stability [102, 103], such as walking on uneven surfaces [104], navigating crowds, and performing activities that require frequent direction changes or responding to unexpected perturbations. From a rehabilitation perspective, these findings highlight the need for a comprehensive approach to balance assessment and training, in which clinicians integrate traditional measures with dynamic wobble-board assessments to obtain a more complete understanding of each patient’s balance control.

These results outline a practical framework for device design and measurement. By distinguishing proactive regulation of excursion magnitude from emergent temporal dynamics, wobble boards should incorporate features that differentially challenge each control component (e.g., adjustable instability, tunable mechanical properties). As we have shown, geometric design parameters—axis alignment (ML/AP/multidirectional), pivot height, and base curvature—can be tuned [105] to target spatial versus temporal control for balance training and rehabilitation. Outcome quantification should extend beyond traditional CoP and CoP–CoM metrics to include parameters that index the persistence of excursion magnitudes and the randomness of cycle durations, enabling finer-grained assessment of how spatial and temporal control evolve with training. Embedding these metrics in intervention protocols allows objective progress tracking and standardized benchmarking of wobble-board–based rehabilitation. Aligning equipment design with metric-driven evaluation can improve intervention precision by enabling individualized prescriptions (e.g., instability level, axis alignment), adaptive progression based on objective thresholds (e.g., shifts in persistence or randomness indices), and quantifiable dose–response tracking across sessions—thereby supporting evidence-based decision-making, facilitating scalable implementation in clinical and community settings, and, ultimately, reducing fall risk.

While these findings establish the construct validity and feasibility of the WB metrics in $N\!=\!29$ healthy young adults, their generalizability to older adults and neurological populations remains to be determined. Future work will (*i*) recruit participants at elevated fall risk to test whether the proactive–emergent division of control (persistent $H$ for excursion magnitudes; near-random $H$ for cycle durations) holds across aging and pathology; (*ii*) incorporate both ML and AP instability—including coupled/multidirectional bases—to characterize axis-specific and coupled control strategies; and (*iii*) evaluate clinical utility via a metric-guided intervention. In the latter, a progressive, instability-graded wobble-board program will target proactive excursion regulation with real-time feedback, with pre-registered hypotheses that changes in the WB metrics predict improvements on standard outcomes (Mini-BESTest, Berg Balance Scale, Timed Up-and-Go, reactive stepping, 10-m gait speed [106–110]) and transfer to uneven-surface walking, prospective fall incidence, and daily life mobility captured by wearables. Together, these steps will determine whether the identified signatures are not only mechanistically informative but also actionable for rehabilitation and fall prevention.

## Data Availability

The dataset used to support the present results is available as Dataset_S1.csv in the Electronic Supplementary Material.
